# Development of a Short Instrument for Measuring Health-Related Quality of Life in Oncological Patients for Clinical Use: Protocol for an Observational Study

**DOI:** 10.2196/17854

**Published:** 2020-07-29

**Authors:** Theresa Schrage, Mirja Görlach, Christian Stephan Betz, Carsten Bokemeyer, Nicolaus Kröger, Volkmar Mueller, Cordula Petersen, Andreas Krüll, Holger Schulz, Christiane Bleich

**Affiliations:** 1 Department of Medical Psychology University Medical Center Hamburg-Eppendorf Hamburg Germany; 2 Department of Otolaryngology University Medical Center Hamburg-Eppendorf Hamburg Germany; 3 Medical Clinic and Polyclinic University Medical Center Hamburg-Eppendorf Hamburg Germany; 4 Department of Stem Cell Transplantation University Medical Center Hamburg-Eppendorf Hamburg Germany; 5 Department of Gynecology University Medical Center Hamburg-Eppendorf Hamburg Germany; 6 Department of Radiotherapy and Radiation Oncology University Medical Center Hamburg-Eppendorf Hamburg Germany

**Keywords:** patient-reported outcomes, health-related quality of life, oncology, psychometric validation, mixed methods study

## Abstract

**Background:**

Cancer patients often suffer from the physical and psychological burden of their disease and its treatment. This is frequently insufficiently identified and addressed in clinical practice. In the context of improving patient-centered care in oncological patients, patient-reported outcomes (PROs) represent an important addition to current routine care. So far, available PRO questionnaires for cancer patients are unsuitable for routine procedures due to their length and complexity.

**Objective:**

This study aimed to develop and psychometrically test a short questionnaire to measure health-related quality of life (HrQoL) in cancer patients for use in routine care.

**Methods:**

This observational study consists of two parts: (1) a qualitative study to develop a short questionnaire measuring HrQoL and (2) a quantitative study to psychometrically test this questionnaire in five oncological departments of a comprehensive cancer center. In part 1 of the study, semistructured interviews with 28 cancer patients, as well as five focus groups with 22 clinicians and nurses, were conducted to identify clinically relevant dimensions of HrQoL. The identified dimensions were complemented with related dimensions from empirical studies and reviewed via expert discussion. Based on this, a short instrument was developed. In part 2 of the study, the developed questionnaire was tested in cancer in- and outpatients at five participating oncological clinics using additional standardized questionnaires assessing HrQoL and other important PROs. The questionnaire was presented to more than 770 patients twice during treatment.

**Results:**

The project started in May 2017 with recruitment for study phase I beginning in December 2017. Recruitment for study phases I and II ended in April 2018 and February 2019, respectively. After study phase II and psychometrical analyses, the newly developed questionnaire measuring the HrQoL of all cancer entities in routine care was finalized.

**Conclusions:**

With five to six dimensions and one item per dimension, the developed questionnaire is short enough to not disrupt routine procedures during treatment and is profound enough to inform clinicians about the patient’s HrQoL impairments and status.

**Trial Registration:**

Open Science Framework Registries 10.17605/OSF.IO/Y7XCE; https://osf.io/y7xce/

**International Registered Report Identifier (IRRID):**

RR1-10.2196/17854

## Introduction

In addition to morbidity rates, the impact of health-related quality of life (HrQoL) has grown in cancer treatment [1]. Medical research shows a continual improvement in early detection and treatment of cancer [1]. Subsequently, there are more long-term survivors, though patients often suffer from the consequences of cancer and its treatment [2]. Assuming that cancer has become a chronic disease for many patients, the HrQoL of cancer patients needs further attention in routine procedures [3].

Cancer patients’ role (eg, in a family or group of friends), their relationship to their spouse, and their social life can be affected by psychological impairments [4,5]. Also, the perceived existential threat to the integrity of patients can have a psychological impact [4]. As a consequence, psychosocial distress, as well as psychological comorbidities (ie, anxiety and depressive disorders) can occur or be amplified during or after treatment [6].

Symptoms of cancer are more or less distinct, depending on the cancer entity [7]. Prostate carcinoma, for example, depicts a symptom-free course for months or years in most cases [8]. On the other hand, pituitary tumors can affect the life of the person immensely [9]. Cancer patients often experience severe side effects due to their treatment such as pain, fatigue, weak immune system, indigestion, sexual dysfunction, nausea, or hair loss [10]. Correspondingly, the negative impact on the patient’s HrQoL can be significant [5], which emphasizes the importance of focusing on HrQoL in clinical practice and research.

In the 1990s, HrQoL was established as an essential part of cancer treatment, and since then it has existed alongside disease-related outcomes [3]. Furthermore, HrQoL can be used to evaluate treatment in cancer patients [11]. At present, HrQoL is used as a parameter for benefit-cost analysis [12], assessing which limitations in quality of life can be endured in exchange for prolonged life. HrQoL proves to be an aspect of patient-reported outcomes (PROs) since information given by the patients themselves [12] is the best source from which state of health can be adequately assessed [11]. This highlights the importance of integrating PROs, and thus HrQoL, into patient-centered care since they have been proven to be more reliable than the clinician’s assessment; they can also aid with earlier symptom identification [13]. Therefore, PROs should be implemented into clinical practice to improve screening of distress, optimize treatment, and measure quality of care [14-20].

The question that is raised is whether HrQoL impairment in a patient is taken into account in cancer treatment. Time is a scarce resource for medical staff and has to be spent carefully [21]. Although patient-centered care, including HrQoL, is eminent and part of the German National Cancer Plan [22], there is currently no sufficient resource-oriented procedure to measure, analyze, interpret, or act upon HrQoL data [23]. Even though there already exists questionnaires to measure HrQoL in oncological patients, the complexity and time-consuming nature of these assessments prevent use in clinical routine. An example of a PRO questionnaire is the European Organization for Research and Treatment of Cancer Quality of Life Questionnaire-C30 (EORTC QoL-C30) [24]. It is a highly recommended tool for the measurement of HrQoL in cancer patients [25]. Since it comprises eight scales, several additional single items, and disease-specific modules, it is not practical for use during routine procedures [26]. Oncological patients are generally older and should be questioned several times over the course of treatment; therefore, a long questionnaire like the EORTC QoL-C30 depletes more capacity than available for both patients and practitioners [27]. These difficulties also occur with other instruments (eg, Functional Assessment of Cancer Therapy [Fact-G] [28], World Health Organization Quality of Life [WHOQOL] [29]) and result in insufficient integration of PRO measurements in clinical routine procedures [27].

This issue can be improved by implementing measurements that are shorter, more adaptive, and prompt action. For example, the Distress Thermometer (DT) is short, which makes it a more applicable instrument [30]. Developed by the National Comprehensive Cancer Network (NCCN), this screening tool determines the type and extent of psychosocial distress in oncological patients. Though it does not measure all aspects of HrQoL, it is often used in clinical practice to screen for need of support and shortages in a patient’s HrQoL. It consists of an 11-step analogue scale from 0 to 10 to measure distress, and a list of problems. However, the NCCN-DT is only used in screening procedures [30] and misses relevant dimensions of HrQoL such as physical complaints and autonomy [31]. Including its list of problems, the NCCN-DT amounts to 36 items, which is a problematic length for a questionnaire used in routine care [27]. Due to its high feasibility [30], however, the NCCN-DT is able to function as a first step in the development of a reliable and adequate instrument in assessing HrQoL in oncological patients.

Hence, there are no suitable short instruments to measure HrQoL in oncological patients for use in clinical routine care. The present study is the first part of a larger project that ultimately targets the implementation of PROs in cancer care clinical practice at the University Medical Center Hamburg-Eppendorf. The aim of this study is to develop a psychometrically validated short instrument with six visual analogue scales with one item each, for repeated measurement of HrQoL of oncological in- and outpatients generalized for all cancer entities. Use of the new instrument in clinical practice is expected to improve patient-centered care, as well as data management for continuous health care research.

## Methods

### Design

**Figure 1:**
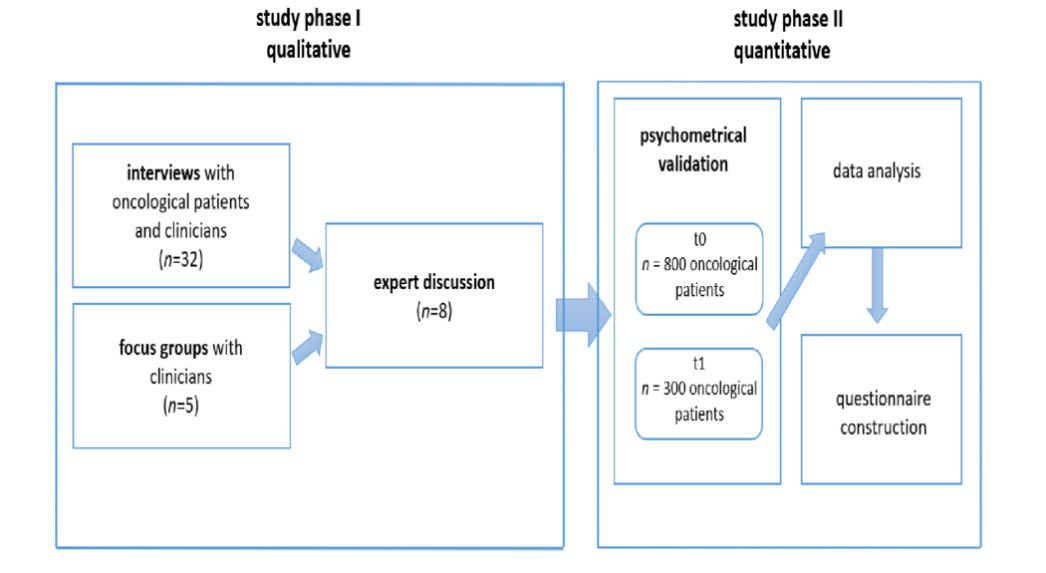
Study design.

In order to develop a validated and reliable instrument, an observational study with a mixed methods design was conducted (Figure 1). The study consists of two phases, making use of qualitative and quantitative data and analyses. Recruitment of study participants took place at the University Medical Center Hamburg-Eppendorf*,* where the developed instrument will also be implemented. The implementation process is outlined elsewhere [32]. Inclusion criteria for patients are a cancer diagnosis, sufficient language skills in German, and no cognitive or verbal impairments in providing information and giving informed consent.

#### Study Phase I

The aim of study phase I is to develop five to six relevant dimensions of HrQoL for cancer patients. For this purpose, interviews with oncological patients (n=28) were conducted. To facilitate further discussion and exchange, five focus groups with oncologists, oncological nurses, and psychologists on relevant dimensions of HrQoL were undertaken. Patients as well as clinicians were asked to evaluate the HrQoL dimensions in general and in regard to cancer treatment. Since senior clinicians could not be present at the focus groups, interviews (n=4) were conducted instead.

The outcome of attained qualitative data was presented to a group of eight experts for discussion. Psychooncologists, oncologists, patient representatives (leaders of self-help groups), quality of life scientists, staff nurses, a quality management representative, and a health insurance representative were included. On the grounds of these outcomes and of the current state of research, the dimensions for the instrument were identified. Items were phrased and discussed among project group members. Wording, meaning, and overall feasibility of the items were verified by means of a survey of health care researchers (n=5). This phase began in December 2017 and ended in April 2018. For additional feasibility testing, a pilot study was carried out. Three patients were asked to evaluate the newly developed instrument by using the reading-out-loud technique. Since no requests for modification of the instrument were voiced, the instrument was readied for psychometrical testing.

#### Study Phase II

To psychometrically test the developed questionnaire, oncological in- and outpatients with different cancer diagnoses and stages of disease were asked to participate. The questionnaire was presented to the subjects in paper-pencil format. Furthermore, medical data were retrieved from the Soarian Clinicals patient documentation system of the University Medical Center Hamburg-Eppendorf. A pilot run was conducted in May 2018. Three patients were asked to provide their opinion using the reading-out-loud technique in order to assess the comprehensibility and feasibility of the questionnaire. The statistical survey started in June 2018 and ended in February 2019.

### Cooperation Partners

Recruitment of patients in study phases I and II was carried out in cooperation with the Medical Clinic and Polyclinic, the Department of Stem Cell Transplantation, the Department of Gynecology, the Department of Radiotherapy and Radiation Oncology, and the Institute and Polyclinic for Medical Psychology.

### Recruitment and Procedures

#### Study Phase I

Potential patients to be questioned were pointed out by staff. The appointed patients were asked to participate and interviewed by research staff. We planned to conduct 28 interviews with oncological patients; four additional interviews with senior clinicians were conducted. Medical staff and external experts were approached and asked to participate. After written consent, they were invited to either an interview, a focus group, or a summarizing discussion. We planned to conduct five focus groups and one expert discussion.

#### Study Phase II

Potential inpatients to be questioned were pointed out by staff in the outpatient departments; oncological patients were addressed directly by research staff. Inpatients were questioned twice, once at the beginning of their treatment and then again 3-7 days later. The interval of time for questioning outpatients differed due to practice procedures. They were asked to participate once during their treatment and again 1 week later.

### Patient and Clinician Involvement

Patients and clinicians were not involved in the study design. Patients first became involved during study phase I when they were interviewed regarding their HrQoL appraisal. Clinicians were involved in the recruitment process and execution of the study. In study phase I, chief physicians were asked to support recruitment of clinicians by asking their staff to participate. For study phases I and II, clinicians were asked to point out potential patients to be questioned. Patients were selected on the basis of assessments made by the clinicians. For example, severe pain or low responsiveness in some cases prevented patients from participating. In order to assess the comprehensibility and feasibility of the questionnaire, three patients were asked to give their opinion using the reading-out-loud technique (pilot study).

### Measurements and Outcomes

#### Study Phase I

A semistructured interview guide was developed based on Helfferich [33], asking one main question concerning relevant dimensions of HrQoL to be assessed in routine care. An example of such a question is “If you imagine that your current doctor asks you about your quality of life, physical and mental stress, what would be important for you? / What should not be forgotten?”. For focus groups, a focus group guide was developed based on Barbour [34], including the same main question used in the interview guide.

#### Study Phase II

To test the validity and reliability of the developed questionnaire, a series of established standardized measurements were included in the quantitative survey. In addition to the developed questionnaire, sociodemographic and standardized questionnaires assessing HrQoL (Fact-G [28]), Distress Thermometers [30], Short Form 8 Health Survey (SF-8) [35]), dignity (German version of the Patient Dignity Inventory [PDI-G] [36]), as well as depressive and anxiety symptoms (Patient Health Questionnaire-4 [PHQ-4] [37]), were included for psychometric purposes. These standardized questionnaires were included to validate the newly developed questionnaire. The priority objective of study phase II is a reduction to five to six HrQoL dimensions with one item each. The final result of this study phase is a psychometrically validated short instrument to assess HrQoL in cancer patients for use in routine clinical practice.

### Data Analysis

#### Qualitative Analysis

Interviews, focus groups, and expert discussion were carried out by scientists, recorded, and afterwards transcribed by study staff. The qualitative data was structured via the software program MAXQDA 10 and analyzed using qualitative content analysis based on Mayring [38]. Within the procedure of analyzing the data, deductive-inductive category application was used: deductive main categories (generated through literature research) and inductive subcategories (derived from the qualitative text analysis of the interviews and focus groups). Quality criteria to be examined for the qualitative content analysis are interrater reliability and communicative validation.

#### Quantitative Analysis

For study phase II data, an exploratory structural equation model (principal component analysis) was planned to be computed via SPSS (IBM). Reliability as well as validity (internal consistency, construct validity, content validity, criterion validity, correlations, and responsiveness) of the instrument were assessed through item and scale analysis. Missing data were compensated for by an expectation–maximization algorithm [28]; in cases of missing data of more than 30% per case, exclusion of data was carried out. Transformations of data were only applied if required by the data structure (ie, nonnormality of residuals). Exclusion of data due to systematic bias or false statement was not necessary. Further exploration of the data to look for unexpected differences or relationships were undertaken using subgroup analysis.

### Sample Size and Power

Confirmative models allowed for the determination of an appropriate sample size; based on Monte Carlo study results, for a power of 80%, a sample size of at least 460 patients is needed [39]. Calculating a dropout rate of 40%, a minimum number of 770 addressed patients ensures enough data will be obtained.

### Ethics and Dissemination

The intention of this project is to improve psychosocial care for cancer patients in routine clinical practice. Patients and health care professionals were asked to participate by joining focus groups and interviews and by completing questionnaires. The written survey methodology does not involve direct intervention in medical procedures. We expect no risks or disadvantages for patients, although one potential but unlikely stressor may arise as participants confront the shortcomings of their quality of life. This could have a negative influence on adjustment to and handling of the illness. However, the participating clinics of this project already offer psychooncological support, which is also available for the patients in this study. A written informed consent is mandatory for participation in the study. Patients interested in the study were informed about the voluntary nature of participation and the possibility to refuse or discontinue participation at any time without any negative consequences. Additionally, patients were informed about the study’s aims and personal risks. For further questions concerning the study, the contact details of study assistants were provided. The study received approval by the ethics committee of the Medical Association of Hamburg (reference number: PV5636).

The project started in May 2017 and is planned for 36 months. In December 2017, the first interviews were carried out. Qualitative data of the first study phase were evaluated by April 2018. Quantitative data collection was conducted from June 2018 to February 2019 with subsequent analysis of the obtained data.

Regarding the dissemination plan, two further publications regarding the development of the short instrument for cancer patients will be published (eg, one paper on study phase I and one paper on study phase II).

## Results

This project is funded by Innovationsfond des Gemeinsamen Bundesausschusses (grant number: 01VSF16024). Recruitment was completed in February 2019.

So far, 32 interviews, 5 focus groups, and 1 expert discussion have been conducted. On the grounds of the qualitative analyses of the obtained material, six dimensions with 10 items were identified. After study phase II (n=630) and psychometrical analyses, the newly developed questionnaire (HELP-5 [Hamburger Inventar zur Erfassung von Lebensqualität bei onkologischen Patienten]; English translation: Hamburg Inventory for Measuring Quality of Life in Oncological Patients), comprising five dimensions with one item each to measure HrQoL in all cancer patients in routine care, was finalized. The first results of both study phases are expected to be submitted for publication by the end of 2020.

## Discussion

HrQoL is of vast importance for cancer patients. In the context of improving oncological patient-centered care, continuous PRO monitoring represents an important addition to current routine care to identify and address patients’ needs [40,41]. As patients are highly affected by their disease and its treatment, tools for measuring HrQoL need to be a permanent feature of routine care. At present, instruments measuring HrQoL do not match the requirements of routine care (ie, brevity, adaptiveness, simplicity). The questionnaire developed in this study should be able to meet the above-mentioned requirements. With five to six dimensions and one item per dimension, the developed questionnaire is short enough to not disrupt routine procedures during treatment but is profound enough to inform clinicians about the patient’s impairments and course concerning HrQoL.

## Appendix

Multimedia Appendix 1 Funding report.

## Abbreviations

DT: Distress Thermometer
EORTC QoL-C30: European Organization for Research and Treatment of Cancer Quality of Life Questionnaire-C30
Fact-G: Functional Assessment of Cancer Therapy
HELP-5: Hamburger Inventar zur Erfassung von Lebensqualität bei onkologischen Patienten (Hamburg Inventory for Measuring Quality of Life in Oncological Patients)
HrQoL: health-related quality of life
NCCN: National Comprehensive Cancer Network
PDI-G: Patient Dignity Inventory—German version
PHQ-4: Patient Health Questionnaire-4
PRO: patient reported outcome
SF-8: Short Form 8 Health Survey
WHOQOL: World Health Organization Quality of Life
