# Supplementary material for: Development of a Short Instrument for Measuring Health-Related Quality of Life in Oncological Patients for Clinical Use: Protocol for an Observational Study
Source: JMIR Res Protoc. 2020 Jul 29;9(7):e17854. doi: 10.2196/17854 (PMC7424483; doi:10.2196/17854)
Supplement: Multimedia Appendix 1 [file resprot_v9i7e17854_app1.pdf]

Gemeinsamer Bundesausschuss
Innovationsausschuss

Innovationsausschuss beim G-BA, Postfach 12 06 06, 10623 Berlin

Universitätsklinikum Hamburg-Eppendorf
Herrn Prof. Dr. Dr. Uwe Koch-Gromus
Martinistraße 52
20246 Hamburg

Besuchsadresse:
Wegelystr. 8
10623 Berlin

Ansprechpartner/in beim DLR-PT:
Dr. Jochen Ziegelmann

Telefon:
+49 30 67055-199

Telefax:
+49 228 3821-1257

E-Mail:
jochen.ziegelmann@dlr.de

Datum:
16. Februar 2017

Förderbescheid

Förderung aus Mitteln des Innovationsfonds zur Förderung von Versorgungsforschung (§ 92a Abs. 2 SGB V) für das Projekt:
„PRO-ONK ROUTINE - Entwicklung eines Kurzinstruments zur Messung gesundheitsbezogener Lebensqualität bei Krebspatienten und Analyse der Implementierung“
Förderkennzeichen: 01VSF16024

Ausführende Stelle: Universitätsklinikum Hamburg-Eppendorf – Stiftungslehrstuhl Klinische Versorgungsforschung – Institut und Poliklinik für Medizinische Psychologie

Ihr Antrag vom 24. August 2016
mit Ergänzungen vom 2. Dezember 2016 (Erklärungen im Original) und 10. Dezember 2016 (E-Mail)

Sehr geehrter Herr Professor Koch-Gromus,

mit dem vorliegenden Förderbescheid wird aufgrund der Entscheidung des Innovationsausschusses vom 24. November 2016 die folgende Projektförderung aus Mitteln des Innovationsfonds gewährt.

I. Genehmigung der Europäischen Kommission

Gemäß Nr. 2.1 des Unionsrahmens für staatliche Beihilfen zur Förderung von Forschung und Entwicklung und Innovation („FuEuI-Rahmen“ - Mitteilung der Kommission vom 27. Juni 2014, ABl. EU C 198/1) sind Einrichtungen für Forschung und Wissensverbreitung („Forschungseinrichtungen“) und Forschungsinfrastrukturen Empfänger staatlicher Beihilfen i. S. v. Artikel 107 Absatz 1 AEUV, wenn sie unternehmerisch handeln. Der Unternehmenscharakter hängt nicht von der Rechtsform (öffentlich-rechtlich oder privatrechtlich) oder dem wirtschaftlichen Charakter (gewinnorientiert oder nicht) ab. Entscheidend ist vielmehr, ob die Forschungseinrichtung eine wirtschaftliche Tätigkeit ausübt, d. h. ob sie auf einem bestimmten Markt Produkte oder Dienstleistungen anbietet. Nichtwirtschaftliche Tätigkeiten sind im Allgemeinen primäre Tätigkeiten von Forschungseinrichtungen und Forschungsinfrastrukturen (z. B. die Ausbildung von mehr oder besser qualifizierten Humanressourcen; unabhängige Forschung und Entwicklung zur Erweiterung des Wissens und des Verständnisses; weite Verbreitung der Forschungsergebnisse auf nichtausschließlicher und nichtdiskriminierender Basis; Tätigkeiten des Wissenstransfers). Übt

ein und dieselbe Forschungseinrichtung sowohl wirtschaftliche als auch nichtwirtschaftliche Tätigkeiten aus, fällt die öffentliche Finanzierung der nichtwirtschaftlichen Tätigkeiten nicht unter Artikel 107 Abs. 1 AEUV, wenn die nichtwirtschaftlichen Tätigkeiten und ihre Kosten, Finanzierung und Erlöse klar voneinander getrennt werden können, sodass keine Gefahr der Quersubventionierung der wirtschaftlichen Tätigkeiten besteht. Es ist deshalb im Einzelfall festzustellen, ob eine Forschungseinrichtung eine wirtschaftliche oder eine nichtwirtschaftliche Tätigkeit ausübt:

Das Universitätsklinikum Hamburg-Eppendorf handelt im nichtwirtschaftlichen Bereich (Ziffer 2.1 Nr. 19 des Unionsrahmens). Die Förderung des o. a. Projekts bedurfte keiner Genehmigung durch die Europäische Kommission.

II. Förderzweck, Umfang der Förderung und Zahlungsplan

Aufgrund Ihres Antrags vom 24. August 2016 mit o. a. Ergänzungen wird Ihnen gemäß § 92a Abs. 1 SGB V sowie der Verfahrensordnung des Innovationsausschusses beim Gemeinsamen Bundesausschuss für die Zeit

vom 1. April 2017 bis 31. März 2020 (Förderzeitraum)

im Rahmen einer **Vollfinanzierung** ein nicht rückzahlbarer Förderbetrag von

bis zu **487.146,00 €**
(in Buchstaben: Vier-acht-sieben-eins-vier-sechs-Komma-null-null Euro),

höchstens jedoch in Höhe der förderfähigen Ausgaben bewilligt.

Die Fördermittel sind zweckgebunden und dürfen nur für die im Förderzeitraum verursachten Ausgaben für das o. a. Projekt abgerechnet werden.

Die Bewilligung setzt voraus, dass die Gesamtfinanzierung des Projekts gesichert bleibt.

Der als Anlage F beigefügte Finanzierungsplan ist als Bestandteil des Förderbescheids verbindlich.

Es ist zu beachten, dass im Finanzierungsplan Mittel in Höhe von 6.000,00 € für die Reiseausgaben der Experten aus der Position Reisen in die Position sonstige Sachausgaben verschoben wurden. Es handelt sich hierbei nicht um Reiseausgaben der Projektmitarbeitenden.

III. Nebenbestimmungen

Bestandteil dieses Förderbescheids sind die beigefügten Allgemeinen Nebenbestimmungen des Innovationsausschusses beim Gemeinsamen Bundesausschuss für Förderungen aus dem Innovationsfonds (ANBest-IF, Anlage N) sowie die nachstehenden besonderen Nebenbestimmungen:

1. Bedingungen für die Wirksamkeit des Förderbescheids (Auflagen)*

falls aufschiebende Bedingung zutreffend:

Die Förderung wird mit der aufschiebenden Bedingung (§ 32 Abs. 2 Nr. 2 Zehntes Buch Sozialgesetzbuch, SGB X) bewilligt, dass **spätestens bis zum 15. März 2017** folgende Auflagen in Abstimmung mit dem DLR Projektträger erfüllt werden:

- Vorlage einer aktualisierten und detaillierteren Zeitplanung zur Fallzahlerreichung entsprechend dem beigefügten Vordruck (Anlage P),

- Vorlage einer nachvollziehbaren und widerspruchsfreien Darstellung von Methodik, Fallzahlplanung und Kontrollgruppe des Studiendesigns,
- Vorlage einer Bestätigung, dass die Verwendung des Instruments nach Abschluss der Förderphase an die Regelungen der ANBest-IF (Anlage N) gebunden ist.

Die Unterlagen sind rechtsverbindlich unterschrieben an den DLR Projektträger zu senden. Dieser Förderbescheid wird erst dann bestandskräftig, wenn die positive Prüfung der genannten Unterlagen durch den Förderer bestätigt wurde. Hierzu wird ein entsprechender Änderungsbescheid erstellt.

2. Meilensteinplan

Der Förderempfänger hat bis zum **15. März 2017** einen Meilensteinplan für das Projekt entsprechend dem beigefügten Vordruck (Anlage M) vorzulegen.

In der bisherigen Meilensteinplanung ist der Endbericht als Meilenstein aufgeführt. Der Schlussbericht im Zusammenhang mit dem Verwendungsnachweis ist dem DLR Projektträger erst nach Abschluss des Projekts vorzulegen. Daher ist der Endbericht ggf. nicht Teil der Meilensteinplanung. Dies ist bei der Aktualisierung des Meilensteinplans zu berücksichtigen.

3. Mittelsperre

Es werden insgesamt **3.300,00 €** gesperrt, davon entfallen auf die einzelnen Positionen des Gesamtfinanzierungsplans:

Sachausgaben (Sonstige Sachausgaben): 3.300,00 €
Die Fördermittel von 3.300,00 € für 10 Tablet-PCs werden aufgrund fehlender Kalkulationsgrundlage und fehlender Erläuterungen gesperrt. Die Notwendigkeit der Tablet-PCs ist näher zu erläutern und durch entsprechende Kalkulationen zu ergänzen. Für die Entsperrung der Mittel sind ein formloser, rechtsverbindlich unterschriebener Antrag mit den o. a. Ergänzungen zur Prüfung vorzulegen.

Gesperrte Mittel des Innovationsfonds können nicht ausgezahlt werden. Von der Sperre betroffene Ansätze sind von der Austauschbarkeit zugunsten anderer Positionen des Finanzierungsplans ausgeschlossen. Die Mittelsperre erfolgt unter dem Vorbehalt einer Kürzung der Mittel oder eines Abbruchs des Projekts. Nach positiver Prüfung wird über die Entsperrung der Mittel durch einen schriftlichen Änderungsbescheid entschieden.

4. Datenschutz

Zur Sicherung des Datenschutzes sind rechtzeitig die erforderlichen datenschutzrechtlichen Maßnahmen zu treffen und in Zweifelsfällen die für die Datenschutzkontrolle zuständigen Stellen (im öffentlichen Bereich die Landesbeauftragten für den Datenschutz und die Bundesbeauftragte für den Datenschutz und die Informationsfreiheit, im Übrigen die betrieblichen Beauftragten für den Datenschutz) einzuschalten.

5. Ethische Richtlinien

Bei der Durchführung von Untersuchungen am Menschen und/oder der Gewinnung bzw. Verwendung von menschlichem Probenmaterial im Rahmen dieses Projekts sind die Empfehlungen der Deklaration von Helsinki sowie die Richtlinien des CIOMS (Council for International Organization of Medical Sciences) und der WHO (World Health Organization): „Proposed International Guidelines For Biomedical Research Involving Human Subjects“ in den jeweils geltenden Fassungen einzuhalten.

6. Ethikvotum

Vor Beginn der Untersuchungen am Menschen und/oder der Gewinnung bzw. Verwendung von menschlichem Probenmaterial ist das uneingeschränkt positive Votum der zuständigen Ethik-

kommission vorzulegen. Falls diese ein Votum nicht für erforderlich hält, ist eine entsprechende Erklärung der Ethikkommission vorzulegen.

7. Widerrufsvorbehalt
Dieser Bescheid kann in einem der folgenden Fälle widerrufen und die Förderung ganz oder teilweise eingestellt werden (Widerrufsvorbehalt nach § 32 Abs. 2 Nr. 3 in Verbindung mit § 47 Abs. 1 Nr. 1 SGB X):

- im Fall, dass der Förderzweck nicht zu erreichen ist,
- in den Fällen einer Mittelsperre für Einzelansätze des Finanzierungsplans,
- in den Fällen einer nicht fristgerechten bzw. unvollständigen Vorlage von Nachweisen,
- aus zwingenden Gründen.

8. Nachweis der Verwendung
Für den Nachweis der Verwendung gelten die Regelungen der Nr. 14 ANBest-IF. Die entsprechenden Vordrucke werden dem Förderempfänger zu gegebener Zeit zur Verfügung gestellt. Für die Belegliste ist der Vordruck nach Anlage B zu verwenden.

9. Projektveranstaltungen
Der Förderer ist über wichtige Termine bzw. Veranstaltungen, Newsletter usw. zu informieren bzw. einzuladen. Auf Verlangen ist jederzeit Auskunft über Art, Umfang und Erfolg der durchgeführten Maßnahmen zu geben.

10. Auszahlung der Fördermittel
Die Fördermittel werden auf Anforderung des Förderempfängers entsprechend der Nr. 7 ANBest-IF ausgezahlt. Projektbezogene förderfähige Ausgaben, die nach Förderbeginn entstanden sind, können später mit der Zahlungsanforderung zur Abrechnung eingereicht werden.

Eine Auszahlung von Fördermitteln kann erst erfolgen, wenn der Förderbescheid nach Ablauf der Rechtsbehelfsfrist bestandskräftig geworden ist, die Bedingungen für die Wirksamkeit des Förderbescheids (Auflagen) gemäß Punkt III. 1 dieses Förderbescheids erfüllt worden sind und die Eingangsbestätigung (Vordruck nach Anlage E) vorgelegt wurde. Der Förderempfänger kann die Bestandskraft des Bescheids vorher herbeiführen, wenn er auf den Rechtsbehelf verzichtet (Vordruck nach Anlage E).

Für die Erstellung der Zahlungsanforderung ist der Vordruck nach Anlage Z zu verwenden und Anlage H zu berücksichtigen. Für den mit der Zahlungsanforderung vorzulegenden Statusbericht (siehe Nr. 7 ANBest-IF) ist der Vordruck nach Anlage S zu verwenden.

Zusätzlich ist mit jeder Zahlungsanforderung über den aktuellen Stand der Fallzahlerreichung für das Projekt anhand des beigefügten Vordrucks (Anlage P) zu berichten.

11. Rückzahlungen
Rückzahlungen von Fördermitteln sowie ggf. Zinsen sind unter Angabe des Förderkennzeichens 01VSF16024 auf das nachstehende Bankkonto zu überweisen:

Kontoinhaber:	Gemeinsamer Bundesausschuss
Bank:	Deutsche Apotheker- und Ärztebank eG
IBAN:	DE69 3006 0601 0004 2118 20
BIC:	DAAEDEDDXXX

12. Beauftragung eines Projektträgers

Als Projektträger für den Innovationsausschuss beim Gemeinsamen Bundesausschuss ist gegenwärtig der DLR Projektträger beauftragt, die Projektförderung im Rahmen des Innovationsfonds abzuwickeln.

Alle die Durchführung und Abwicklung des Projekts betreffenden Vorgänge sind an den Projektträger zu senden:

DLR Projektträger
– Bereich Gesundheit –
Heinrich-Konen-Str. 1
53227 Bonn

<u>Fachliche Betreuung</u>:
Dr. Jochen Ziegelmann
Tel.: +49 30 67055-199
E-Mail: jochen.ziegelmann@dlr.de

<u>Administrative Betreuung</u>:
Axel Weirich
Tel.: +49 228 3821-1838
E-Mail: axel.weirich@dlr.de

IV. Rechtsbehelfsbelehrung

Gegen diesen Bescheid kann binnen eines Monats nach Bekanntgabe Klage erhoben werden. Die Klage ist beim Landessozialgericht Berlin-Brandenburg, Försterweg 2-6, 14482 Potsdam, zu erheben.

Eine **Durchschrift** des Bescheids wird an das Universitätsklinikum Hamburg-Eppendorf – Stiftungslehrstuhl Klinische Versorgungsforschung – Institut und Poliklinik für Medizinische Psychologie per E-Mail übersandt.

Mit freundlichen Grüßen

Prof. Josef Hecken
Dieser Bescheid wurde elektronisch erstellt und trägt daher keine Unterschrift.

Anlagen:

- Allgemeine Nebenbestimmungen des Innovationsausschusses beim Gemeinsamen Bundesausschuss für Förderungen aus dem Innovationsfonds (ANBest-IF), Stand Oktober 2016 — Anlage N
- Eingangsbestätigung und Rechtsbehelfsverzicht — Anlage E
- Finanzierungsplan — Anlage F
- Vordruck Zahlungsanforderung — Anlage Z
- Hinweise zum Ausfüllen der Zahlungsanforderung — Anlage H
- Vordruck Statusbericht zur Zahlungsanforderung — Anlage S
- Vordruck Fallzahlplan — Anlage P
- Vordruck Meilensteinplan — Anlage M
- Vordruck der Belegliste als Anlage zum Verwendungsnachweis — Anlage B
